# Sociocultural and perceived public image of nurses among nursing students: the mediating role of self-concept

**DOI:** 10.1186/s12912-024-01957-2

**Published:** 2024-04-30

**Authors:** Lailani Sacgaca, Eddieson Pasay an, Aida Sanad Alqarni, Petelyne Pangket, Salman Amish Alshammari, Magda Yousif Ramadan, Ameera Khaled Alonezei, Fatmah Ahmed Alamoudi, Insaf Hassan Ahmed Mohammed, Dolores Cabansag, Lizy Sonia Benjamin

**Affiliations:** 1 Department of Nursing, Prince Sultan Military College of Health Sciences, Dhahran City, Saudi Arabia; 2 Nursing Administration Department, College of Nursing, King Khalid University, Abha, Abha City, Kingdom of Saudi Arabia; 3 Department of Medical-Surgical Nursing, College of Nursing, King Khalid University, Abha, Kingdom of Saudi Arabia; 4 Department of Medical-Surgical, College of Nursing, University of Hail, Hail City, Kingdom of Saudi Arabia; 5 Nursing Service Department, King Khalid Hospital, Ministry of Health, Hail City, Kingdom of Saudi Arabia; 6 Department of Nursing, Aldarb University College, Jazan University, Saudi Arabia, Jazan City, Saudi Arabia

**Keywords:** Nurses, Nursing students, Self-concept, Sociocultural, Public image

## Abstract

**Background:**

Studying the public perception of nurses among nursing students is vital in establishing strategic solutions to recruit and retain more students in nursing programs and to contain nurses in the health care system.

**Aim:**

This study aimed to determine the mediating role of self-concept in the relationship between sociocultural and perceived public image of nurses.

**Methods:**

This study employed a correlational approach using convenience sampling of 1390 participants. This study was conducted at six large universities in three regions of Saudi Arabia (central, northern, and eastern). Student nurses in their second to fourth years of study were included as participants, with an 89.7% response rate. Data were collected from January to April 2023.

**Results:**

A significant positive relationship was observed between sociocultural factors and self-concept (*r* = .685, *p* = .0001). In addition, there was a significant positive relationship between self-concept and public image (PI) (*r* = .352, *p* value = 0.0001). Furthermore, there was a significant positive relationship between sociocultural and public image (*r* = .456, *p* = .0001); sociocultural had a direct effect on self-concept (β = 0.324, SE = 0.098, t = 9.429, *p* < .0001) and public image (β = 0.605, SE = 0.038, t = 22.617, *p* < .0001). Furthermore, sociocultural had an indirect effect on public image through self-concept (H6) (β = 0.389, SE = 0.123, t = 12.766, *p* < .0001).

**Discussion:**

The study findings suggest that nursing school programs should take measures to foster a supportive environment that promotes self-concept and public image, while also being mindful of the sociocultural background. This would also open the scope for further research on the matter involving multiple centers.

**Conclusions:**

This study suggests the need for programs to boost self-concept and public image that consider sociocultural influences. These ’findings have crucial implications for student nurses’ social and psychological wellbeing as they improve the understanding of how sociocultural affects self-concept and public image.

## Introduction

The heroic role partaken by nurses during the COVID-19 pandemic as frontliners was pivotal in sustaining the healthcare system [1]. However, notwithstanding the social significance of the profession, the image of nurses remains ambiguous, devalued, and with differing fallacies, which wield negative impacts and stereotypes from the public [2]. Society has incoherently and mistakenly deduced how the nursing profession emerged through education and its evolution from an established scientific body of knowledge and principles [3]. Stereotypes isolating the discipline as a woman’s job and without professional identity, as it is subordinate to medical physicians, have existed throughout history [4, 5]. Nurses were described negatively as being in an inferiorly position and as assistants to physicians [6], perceived as women with lower educational levels [7], and considered incapable of being leaders [8]. These stereotypes and negative characterizations [9] exert undesirable constraints on the creation of positive perceptions of nurses’ image and value [8] recruitment and retention of nursing students [10], and recruitment opportunities [11]. Such a profound and plausible image of nursing is central to the dissipation of erroneous and fallacious public perspectives [12].

Nursing image is a complex concept, considering the various factors that give rise to its emergence [13]. The formation of nursing public images can affect nursing students’ professional identity and behavior towards nursing care [14, 15] suggesting that determining negative perceptions of nurses’ public image early can enhance professional development and boost a positive image. Numerous studies claim that the public image of nurses is perceived negatively [16, 17]. In contrast, a favorable perception of nurses was observed [18]; however, few studies have highlighted the positive perception of nurses’ public image, status, and power as a profession during the COVID-19 pandemic [19–21].

Sociocultural factors are one of the elements that significantly contribute to the development of an unpleasant public perception of nursing [11, 22]. The sociocultural theory claims that human behaviors are sensitive to the intricacies of the social world around them and that the process of learning is influenced by cultural concepts, activities, and artifact [23]. Moreover, the sociocultural subgroups of ethnicity, religion, race, gender, social class, family traditions, age, and peer groups, including individual and group cultures, significantly affect cognition, actions, emotions, decision-making, and other aspects of human life [24].

Self-concept (SC) refers to embodied beliefs and values and how they influence thoughts and behaviors [25]. Consequently, nurses with strong SC can have a good impact on patient care, while those with weak SC could have the opposite effect [26]. One study revealed that continuously nurturing SC at school is important because it helps students become successful in their nursing career [14]. It has also been observed that SC is strongly related to nurses’ retention [27]and [28–30]. It is also shown that the higher the nurses’ professional SC, the lower the work-related burnout. Furthermore, SC is a predictor of personal accomplishment, depersonalization, and emotional exhaustion among nurses [29].

Both sociocultural factors and SC are essential for developing either a positive or negative perception of the public image of nurses and the profession. However, to the best of our knowledge, no study has demonstrated the mediating role of SC in the association between sociocultural variables and nursing students’ perceptions of nurses. For the purpose of developing strategic solutions to attract more students to the nursing profession, keep students enrolled in the nursing programme, and limit the number of nurses in the healthcare system, a thorough investigation of the variables influencing the public view of nurses among nursing students is essential. Additionally, the need for nurses has risen to help address the worldwide shortage of nurses, strengthen the fragile healthcare system that is relentlessly affected by the COVID-19 pandemic and other infectious diseases, and prevent avoidable healthcare crises in the future. In this study, we assumed that sociocultural factors have a direct effect on SC, SC has a direct effect on public image, and there is an indirect sociocultural effect on public image through SC. Therefore, this study aims to determine the mediating role of SC between student nurses’ sociocultural and perceived public image of nurses.

## Methods

### Research design

This study employed a correlational approach using convenience sampling.

### Setting/sample

This study was conducted at six large universities (two in each region) in three regions of Saudi Arabia (central, northern, and eastern). Student nurses in their second to fourth years of study were included using convenience sampling. The participants were included based on the following criteria: (a) voluntary participation, (b) ability to comprehend English, and (c) started nursing as their first program. Initially, 1550 student nurses were invited to participate, and 1390 individuals responded to the questionnaire (89.7%).

### Questionnaires

This study utilized three questionnaires.

#### Nursing image scale

In order to ascertain an individual’s perception of the nursing profession, the Nursing Image Scale (NIS) developed by Özsoy [31]. The original questionnaire and its scale which was published by the original developers was adapted in this current study. The tool includes 28 items with the choices “I agree,” “I partially agree,” and “I do not agree” on a 3-point Likert scale. The minimum and maximum scores were 28 and 84 respectively, with higher values reflecting a positive view of nursing. It comprises three subscales that cover general appearance (1–7), communication (8–13), and occupational and educational attributes (1–28). Cronbach’s alpha coefficient, which was 0.80 and indicated strong internal consistency, was used to assess the dependability of the tool [31].

#### Personal SC questionnaire

This scale assessed the participants’ self-concept. This questionnaire and its scale was developed and published by Goni et al. [32] which is intended to evaluate the following four essential aspects of self-concept: emotional adjustment, self-fulfillment, autonomy, and honesty. On a 5-point Likert scale, the 22 items are assessed, from totally disagree to totally agree. It was composed of 4 subscales including self-fulfillment (1–6 statement), autonomy (7–11 statement), emotional adjustment (12–17 statements), and honesty (18–22 statements). Internal consistency was established using Cronbach’s alpha with a reliability index of 0.85 and an explanatory factorial analysis that explained 52.56%, signifying an acceptable range [32].

The culture questionnaire and its scale was adapted from the original author [5] which was used to seek and gauge participants’ perceptions of their sociocultural worldviews. The tool comprises 28 questions with the following response options, on a 7-point Likert scale: strongly agree, disagree, either agree or disagree, slightly agree, agree, and highly agree. It has nine subscales: assertiveness (15–18 statements), future orientation (19–22 statements), performance orientation (23–24 statements), humane orientation (25–28 statements), power distance (1–3 statements), institutional collectivism (7–9 statements), in-group collectivism (10–11 statements), gender egalitarianism (12–14 statements), and uncertainty avoidance (1–2 statements). Cronbach’s alpha was used to examine reliability, and the results indicated that it was 0.63 for power distance, 0.81 for avoiding ambiguity, 0.81 for collectivism, 0.61 for masculinity, and 0.85 for long-term orientation. The discriminant and convergent validity of the measures were confirmed by a factor analysis employing oblique rotation, which explained 49% of the total variance [5].

As the questionnaire is publicly available for non-commercial and research uses, permission from the original creator was not requested. The three questionnaires underwent content and cultural sensitivity testing as well as validation. Five nursing practitioners and four nursing education professionals served as the validators. As a result, all nine experts were in agreement that each item seemed appropriate for the concept being intended. The instrument’s validity was examined using a pre-test sample of 18 student nurses. The Cronbach’s alpha coefficient for SC, 0.88 for public perception, and 0.94 for sociocultural variables were all calculated.

### Data collection

Data collection began with the permission of the university authorities and clearance from the Institutional Review Board of the Prince Sultan Military College of Health Sciences. Prior to orientation, the point person for each of the large universities posted an invitation for orientation at the students’ lounge. After that, a point person from each university conducted an orientation with the participants to explain the aim of the study, extent of their participation, and their rights, including the right to withdraw, should they feel pressured. After the orientation, the point persons gave the paper questionnaire to the participants and were given ample time to decide whether to participate or not. An informed consent form was attached to the questionnaire and students were instructed to read and sign the informed consent should they understand and willing to participant. Participants who agreed to participate were instructed to answer the questionnaire for at least 20–30 min during their break time. Data were collected from January to April 2023.

### Ethical consideration

The Institutional Review Board of the Prince Sultan Military College of Health Sciences (approval number IRB − 2023-NUR-431) gave its approval to this study dated December 3, 2022. The anonymity, confidentiality, and privacy of each participant were assured. Moreover, informed consent was obtained from all subjects and all methods were carried out in accordance with relevant guidelines and regulations.

### Data analysis

The data was examined using SPSS version 26. Descriptive statistics were represented as frequencies and percentages. To investigate the structural relationships between the variables, a correlational model was used. The construct relationships were investigated and the hypotheses were validated using AMOS 26.

## Results

The majority of participants (85.3%) were 25 years old or younger; most of the participants (97.8%) were women, and the majority (93.7%) were Saudi.

Table 1 presents the relationships between the variables observed in this study. To evaluate the structural model between the variables, correlational matrices between the observed variables were computed. A significant positive relationship was observed between sociocultural factors (C) and SC (*r* = .685, *p* = .0001). In addition, there was a significant positive relationship between SC and public image (PI) (*r* = .352, *p* value = 0.0001). Furthermore, there was a significant positive relationship between sociocultural factors and public image (*r* = .456, *p* = .0001).

**Table 1:** Correlation matrix between the studied variables

	PI	SC	C
PI	1	0.352^**^	0.456^**^
SC	0.352^**^	1	0.685^**^
C	0.456^**^	0.685^**^	1

** Significant at 0.01

C, sociocultural; PI, public image; SC, self-concept

**Fig. 1:**
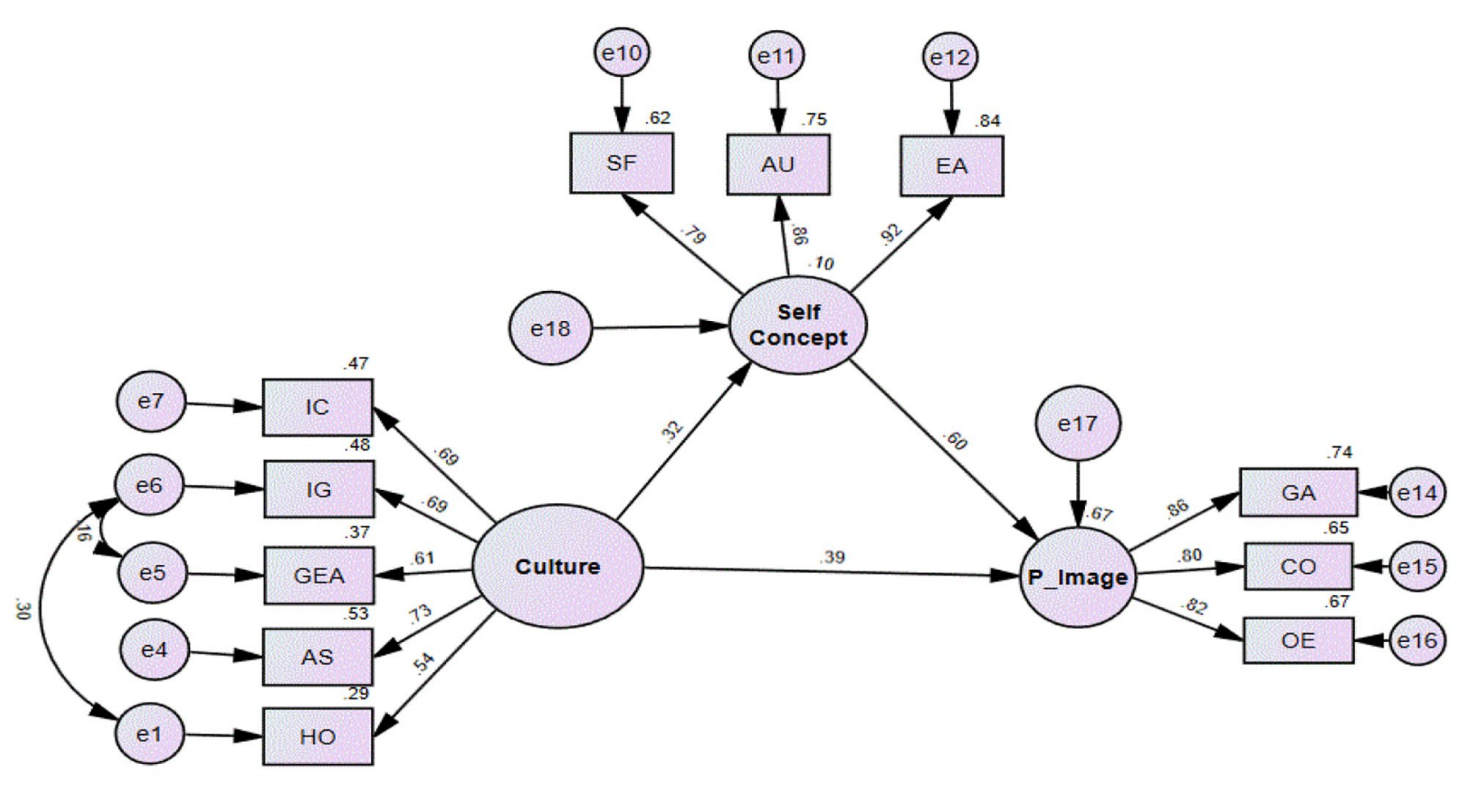
Path analysis model. AS = Assertiveness; IC = Institutional Collectivism; GEA = Gender Egalitarianism; IG = In Group Collectivism; HO = Human Orientations = Self-Fulfillment; AU = Autonomy = Emotional Adjustment; GA = General Appearance = Communication; OE = Occupational and Educational Characteristics

AMOS 26 was applied to test the study hypotheses and look at the generated associations. The results for the path coefficient are displayed in Fig. 1.

Table 2 shows that all three variables have Cronbach’s alpha values above the commonly recommended threshold of 0.7, indicating good internal consistency. Additionally, the Composite Reliability scores for all three variables are higher than the suggested cutoff point of 0.7, indicating good internal consistency. The average variance extracted (AVE) is a convergent validity metric that evaluates the degree to which items on a scale measure the same construct. Furthermore, all three variables have a reliable indicator of convergent validity of an AVE value over the suggested cutoff point of 0.5.

**Table 2:** Cronbach’s alpha, composite reliability, and average variance extracted

	Cronbach’s alpha	CR	AVE
Culture	0.804	0.864	0.561
Public image	0.867	0.919	0.790
Self-concept	0.891	0.932	0.819

AVE, average variance extracted; CR, composite reliability

Table 3 shows that the fit indices suggest a good fit between the data and the hypothesized measurement model, indicating that the proposed model is appropriate.

**Table 3:** Model fit criterion

Measure	ACCEPTABLE FIT	RESULT
CMIN		583.487
SRMR	> 0.05	0.023
GFI	0.90-0.95	0.935
NFI	0.90-0.95	0.932
RFI	0.90-0.95	0.904
IFI	0.90-0.95	0.936
TLI	0.90-0.95	0.91
CFI	0.95-0.97	0.936
RMSEA	0.05- 0.10	0.098

CFI = Comparative fit index; CMIN = Chi-square minimum; GFI = Goodness of fit index: IFI = Incremental Fit Index = Normed Fit Index: RFI = Relative Fit Index RMSEA = Root mean square error of approximation = Standardized Root Mean Squared Residual; TLI = Tucker-Lewis index

Table 4 summarizes the results of the path analysis. The hypothesis is supported by the direct effect of C on SC (H4) (β = 0.324, SE = 0.098, t = 9.429, *p* < .0001), direct effect of SC on PI (H5) (β = 0.605, SE = 0.038, t = 22.617, *p* < .0001), and indirect effect of C on PI through SC (H6) (β = 0.389, SE = 0.123, t = 12.766, *p* < .0001).

**Table 4:** Path analysis results of the different hypotheses

Path of Hypotheses	Path coefficient (β)	Standard Error	T value	Decision
C -> SC (H4)	0.324	0.098	9.429***	Accepted
SC -> PI (H5)	0.605	0.038	22.617***	Accepted
C-> SC -> PI (H6)	0.389	0.123	12.766***	Accepted

*** Significant at 0.01 C, sociocultural; PI, public image; SC, self-concept

## Discussion

In this study, we investigated the mediating role of SC between sociocultural factors and perceived public image of nurses among student nurses. We found a connection, between factors and self-concept indicating that the social and cultural context in which student nurses are immersed greatly impacts their self-perception. A positive self-concept can be nurtured through exposure to influences like positive classroom environments encouraging feedback from teachers and peers and ample opportunities for personal and professional growth [8, 33]. This suggests that self-concept and confidence are intertwined with nurses’ social abilities highlighting the link between aspects (such as relationships) and their self-perception. The significant relationship between factors and self-concept among student nurses has implications for nursing practice underscoring the importance of adopting culturally sensitive approaches to patient care. This study underscores the value of diversity and inclusivity in the nursing field. By embracing cultures nursing students can develop self-concepts that positively impact patient care outcomes. Therefore, it is crucial, for nursing institutions to prioritize creating an environment that fosters competence and celebrates diversity.

There exists a correlation, between self-concept (SC) and public image in relation to student nurses. This suggests that student nurses who hold a view of the image of nursing are more likely to develop a strong professional identity and feel satisfied with their career choices. The positive perception of nursing in the eye can boost the self-assurance and value that student nurses attribute to themselves. It can also contribute to attracting individuals to join the nursing field thereby addressing shortages in this profession. Understanding this connection is vital as it impacts student’s decisions to pursue nursing as a career their performance at work and their commitment to remain in the profession [34]. Factors such as backgrounds and credentials public perceptions of nursing including issues like lack of leadership development and professionalism portrayals in media and online platforms individual patient experiences and collaboration, with other professionals all play unequal roles for various reasons [35]. Nurses should be provided a positive image to combat their negative self-concept, low self-esteem, and negative public perception [9]. Perception of the nursing profession influences resource allocation, field development, work performance, workload, fatigue, and job satisfaction [34]. Therefore, this study contributes to nursing education by creating strategies that support a self-image in students promoting a feeling of self-value and identity, within their profession. In fact, programs that encourage students to develop a healthy sense of self and professional identity should be prioritized in nursing education. This goal can be achieved through the provision of inspirational role models, promotion of nursing’s significance as a profession, and establishment of a welcoming classroom atmosphere. By taking these measures, nursing schools can help their students form a solid professional identity, which, in turn, will improve the quality of care provided and public’s view of nurses.

Furthermore, there is a strong positive correlation between influences and public opinion indicating that aspiring nurses draw inspiration for their personal identities from how society perceives the nursing profession. A positive public perception of nursing could potentially lead to increased interest, in pursuing nursing careers and higher job satisfaction among practicing nurses ultimately benefiting outcomes. Research conducted in Turkey revealed that a positive public image and societal status influenced individuals interest in the nursing field [36]. Another study demonstrated a correlation between nurse’s perceptions of image and their own self esteem [39]. Factors such as media representations and relationships with colleagues also play a role in shaping the perception of nursing as highlighted by research on behaviors experienced by nursing students and its impact on ethical standards [37]. Given that perception affects aspects such as resource allocation, professional growth, employee performance, stress levels and job satisfaction, within the nursing field it is crucial for nurses to uphold a favorable image both externally and internally [13].

In order to tackle the lack of nurses and optimize resource distribution the nursing field should focus on establishing a reputation that reflects its core values, debunking misconceptions and drawing in fresh talent. Moreover, nursing procedures should target the factors causing conflicting perceptions of the profession to enhance nurses effectiveness enhance results and boost perception of nursing. Sociocultural factors have a direct effect on the SC of student nurses, suggesting that personal factors, extrinsic factors in the clinical learning environment, and social support have a substantial effect on student nurses SC [38]. Sociocultural factors have a significant influence on students [39]. Additionally, a study indicated that nurses’ SC influences nursing students’ clinical decision-making [40]. Self-compassion and professional SC acted as mediators in the link between perceived social support and self-esteem in healthcare workers [41]. Previous study also discovered that social support has a big impact on how healthcare professionals perceive themselves. A recent study found that self-compassion and professional SC influence the link between perceived social support and self-esteem among healthcare workers [41]. The same study found that social support significantly influences the self-perception of healthcare professionals. The goal of nursing education is to provide students with a healthy sense of identity and the ability to make sound clinical judgment. To further aid the growth of nurses’ sense of self and clinical decision-making abilities, nursing practice should permit situational awareness, autonomy, and the freedom to make proper decisions.

To address the shortage of nurses and improve resource allocation in the nursing field it is important to focus on building a reputation that reflects the values of nursing dispelling misconceptions and attracting new talent. It is also crucial, for nursing practices to address the factors that contribute to conflicting perceptions of the profession in order to enhance nurses’ effectiveness improve outcomes and enhance the perception of nursing. The social and cultural factors directly impact the self-concept of student nurses indicating that factors external factors in the clinical learning environment and social support significantly influence student nurses’ self-concept. These sociocultural factors play a role in shaping students perceptions. Furthermore, research has shown that nurses self-concept influences decision making among nursing students. Self-compassion and professional self-concept serve as mediators in connecting perceived support with self-esteem among healthcare workers. Previous studies have also highlighted the impact of support on healthcare professional’s self-perception. Recent research has demonstrated that both self-compassion and professional self-concept play a role in linking perceived support with self-esteem among healthcare workers underscoring the importance of support, on healthcare professionals’ perception of themselves. The aim of nursing education is to help students develop a sense of self and the capability to make clinical judgments. To enhance nurses’ self-awareness and clinical decision making skills nursing training should support awareness, independence and the freedom to make choices.

Additionally, there was a direct effect of SC on public image, indicating that student nurses’ perceptions of themselves can directly influence how the public perceives nursing as a profession. Self-esteem of nurses is influenced by both their public image and their own sense of self [42]. If student nurses have a negative self-image, this can result in a negative public perception of nursing, which can negatively impact their self-esteem. This study emphasises how important it is to improve the nursing profession’s reputation and foster a supportive workplace culture in order to raise the standard of nursing care. Ultimately, a positive SC among student nurses can improve the public’s perception of nursing and increase the quality of care provided. Students who have low self-esteem about themselves may project such views onto the public [42]. Student nurses’ personal sense of worth may suffer because of this poor representation of the profession. However, the quality of nursing care can be improved by boosting the profession’s reputation and creating a supportive workplace [34]. Improved patient outcomes and a more positive public opinion of the nursing profession are both associated with nursing students’ positive self-concept [42] The nursing profession may benefit from this, as it may influence policymaking, productivity at work, and commitment to the field. Nursing instructors should emphasize the value of a positive SC among student nurses in order to improve the quality of care delivered. This can be accomplished by encouraging positive self-talk and fostering a positive workplace environment. Moreover, nursing practice can benefit from a greater understanding of the impact of SC on public image.

Through SC, we were able to determine how sociocultural elements, such as education, the workplace, professional aspirations, and conventional social and cultural attitudes, indirectly affect public perception. As such, it all plays a role in shaping the public’s perception of nurses and the profession [43]. Numerous variables affect the public’s perception of nurses, ranging from societal norms to nursing students’ individual identities. A study on nursing students’ perspectives on sociocultural elements in clinical learning found that their perceptions of these factors had a major effect on their sense of self and professional identity [38]. Nurses’ sense of self and professional identity are shaped by several elements, including their professional reputation, workplace, work values, training, and upbringing [42]. While isolation, apathy, and disengagement are significant negative outcomes that can arise from these circumstances [44] it is essential to note that there are additional adverse effects associated with challenges in professional identity formation. Moreover, social isolation and loneliness can result in a lack of motivation, disinterest in activities, emotional detachment, difficulty setting goals, challenges in maintaining relationships, and social withdrawal [45]. Improving nurses’ public profile requires nurses to cultivate a healthy sense of self and a solid sense of professional identity [42]. Positive SC and professional identity development are the fundamental goals of nursing education programs. Mentorship programs, clinical experiences, and academic courses can help instill the sense of value in the minds of these future healthcare professionals. Nursing students can better prepare themselves to combat the negative effects of sociocultural variables on the profession’s public image if they develop a strong sense of professional identity and SC early on. This, in turn, can help improve public perceptions of nurses and their contributions to healthcare. Positive self-perception increases the likelihood of nurses providing high-quality care, experiencing greater job satisfaction, and feeling more confident about their abilities.

### Study limitations

This research had a number of limitations. For instance, it relied on data based on self-reported perceptions, from survey participants. The use of convenience sampling means the results cannot be generalised due to bias. Furthermore, without cross referencing the data each result could only be verified independently. The conclusions drawn were based on a survey conducted in three regions. Given these findings, we recommend conducting a follow up study using a mixed of research methods.

### Implication to nursing education

Developing a learning environment that embraces diversity and encourages self-awareness and positive social connections among students is crucial as shown by the link, between sociocultural aspects and student nurses social competence [46]. Nursing programs should offer courses that support students in building confidence and effective communication skills given the connection between how students view themselves and their professional standing [46, 47]. The importance of nurturing a nursing workforce capable of delivering culturally sensitive care to patients from diverse backgrounds is underscored by the significant correlation between sociocultural factors and public perception among student nurses [48]. Introducing competency training in nursing curricula can help students gain insights into diversity and its impact on their sense of self [49]. According to Jahromi et al. [50] enhancing student nurses’ social competence can enhance their reputation and communication abilities with both patients and healthcare colleagues. The observed indirect impact of factors on perception, through student nurses’ self-image suggests that enhancing cultural competency training can positively influence how students perceive themselves leading to improved professional reputations. To enhance how students view themselves and their reputation and encourage the provision of sensitive care it is essential for nursing instructors to focus on cultural competence training, within nursing education. The public image of nurses has professional and social implications, including their performance in rendering quality care and establishing a positive work environment [9, 34]. Nurses’ professional identity, public image, and SC emanate from the education, values, and ethics of their workplace, and society’s cultural heritage, habits, mores, and traditions.

## Conclusions

We noted significant relationships between factors and self-confidence (SC) as well as between SC and public image. Furthermore, there was a link between factors and public perception. This could be attributed to the impact of factors on SC followed by the influence of SC on how the public perceives individuals. Notably societal factors influenced perception through SC within the group under study. This research underscores the importance of initiatives aimed at enhancing both SC and public image while taking into account influences. The results bear implications, for the mental wellbeing of student nurses as they enhance their comprehension of how societal influences shape SC and public perception.

## Data Availability

No datasets were generated or analysed during the current study.
